# Impact of the COVID-19 pandemic on individuals working in otolaryngology: a cross-sectional survey

**DOI:** 10.1016/j.bjorl.2026.101859

**Published:** 2026-07-22

**Authors:** Charmaine Szalay-Anderson, Benjamin Ewanchuk, Ranjani Somayaji, Derrick R. Randall

**Affiliations:** a University of Alberta, Department of Obstetrics and Gynecology, Edmonton, Alberta, Canada; b University of Calgary, Cumming School of Medicine, Calgary, Alberta, Canada; c University of Calgary, Cumming School of Medicine, Department of Biochemistry and Molecular Biology, Calgary, Alberta, Canada; d University of Calgary, Department of Medicine, Calgary, Alberta, Canada; e University of Calgary, Department of Microbiology, Immunology and Infectious Disease, Calgary, Alberta, Canada; f University of Calgary, Department of Community Health Sciences, Calgary, Alberta, Canada; g University of Calgary, Cumming School of Medicine, Department of Surgery, Section of Otolaryngology - Head and Neck Surgery, Calgary, Alberta, Canada

**Keywords:** Otolaryngology, COVID-19, Safety, Surgery

## Abstract

**Objective:**

The COVID-19 pandemic is caused by the highly infectious and transmissible SARS-CoV-2 virus. Otolaryngology-Head and Neck Surgery (OHNS) practice is at high risk of transmission and subsequently affected by guideline changes and procedure cancellations or postponements. We aimed to gather opinions regarding the pandemic’s personal and professional impact from OHNS professionals globally.

**Study design/Setting:**

A cross-sectional survey was developed and administered using snowball sampling for surgical staff, trainees, and connected allied health workers in OHNS globally in May 2021.

**Methods:**

Item generation was developed via selection of measures from an existing global perspectives survey with additional measures piloted by clinical context experts, service providers, and researchers.

**Results:**

The majority of the 452 total responses were collected from the Americas (68.9%) and identified as female (64.4%). The most commonly reported workplace challenge was fear of illness (21.1%). Respondents generally favoured policies implemented by local health authorities (69.7%) but believed that policies were implemented too late (49.6%). Three main themes surfaced: 1) Unclear guidelines for allied OHNS professionals, 2) Fear regarding COVID-19 transmission due to varied understanding of transmission and PPE adoption, and 3) Resilience of OHNS workers despite continued professional and personal challenges.

**Conclusion:**

Individuals reported generalized fear and frustration in their personal and professional lives due to COVID-19 pandemic. However, they exhibited agility to overcome these challenges. Ongoing support from government and OHNS societies is key to aid health professionals providing essential care.

**Level of Evidence:**

Level 4, cohort cross-sectional survey.

## Highlights

• OHNS and allied health practitioners felt increased risk of coronavirus exposure.
• Practitioners were mostly concerned about transmitting virus to loved ones.
• Practitioners mostly agreed with restrictions imposed but not timing or degree.

## Introduction

The COVID-19 pandemic has undeniably challenged global health care systems, being incomparable to any infectious disease in recent history. Since the first reported case in Wuhan, China, in December 2019, the novel SARS-CoV-2 coronavirus responsible for the COVID-19 pandemic, has spread exponentially worldwide.^1^ Transmission primarily occurs through respiratory droplets in close proximity, such as coughing.^2^ Beyond the rapid transmission of COVID-19, management was further complicated by the virus’s continued evolution.^3^ This ongoing pandemic has required health care practitioners to continually adapt to novel recommendations for both personal and patient safety, all while maintaining high standards in quality patient care delivery.^4^

In the early days of the pandemic, a large proportion of cases were identified in Healthcare Workers (HCWs) related to the occupational spread in healthcare settings.^5^ A total of 1496 COVID-19 related deaths amongst HCWs have been reported in United States,^6^ and 46 deaths have been reported in Canada since the pandemic’s onset.^7^ Moreover, an estimated 115,500 HCWs have died due to COVID-19 between the onset of the pandemic to May 2021 globally.^8^ Given the close interface of patients and healthcare practitioners, there are many junctures in healthcare provision where infections can spread via nosocomial infection.^9^ Nosocomial transmission of SARS-CoV-2 is a clearly identified risk factor to be mitigated in healthcare settings globally.^5^^,^^10^

Several modifications to healthcare delivery were made to mitigate nosocomial spread, including procedures surrounding surgeries.^4^^,^^11^ Surgical and office-based procedures that generate aerosols are considered the highest risk for transmission of SARS-CoV-2, with reports of deaths from operating room-related transmissions. Individuals working in OHNS settings are at particularly high-risk due to the intimate contact with the upper respiratory tract ‒ the reservoir for SARS-CoV-2 ‒ with all procedures, and the potential for aerosol generation.^12^ Amongst the deaths of HCWs early in the pandemic, otolaryngologists and ophthalmologists have been affected at disproportionate rates compared to other surgical fields. Otherwise, previous surveys of concerns of HCWs in OHNS have highlighted higher stress levels due to a high proportion of general anxiety and fear amongst care providers.^13^, ^14^, ^15^ Furthermore, agility in adapting clinical procedures and workflows to minimize the risk of transmission were added sources of stress amongst OHNS professionals.^15^ This was shown in Ireland where a group of otolaryngologists surveyed reported significant anxiety surrounding a lack of control and increased risk of COVID-19 contraction as compared to other healthcare specialties.^13^

Despite representing core practice partners in OHNS, often exposed to similar risk procedures, evaluation of the impact of COVID-19 on OHNS HCWs, such as nurses and administrative staff has thus far been limited. Previous surveys assessing the impact of individuals in OHNS working during the COVID-19 pandemic have been limited to physicians and medical trainees.^13^^,^^16^, ^17^, ^18^ OHNS societies and local bodies have released guidance with regards to optimal Personal Protective Equipment (PPE), disinfection procedures, and a move to conduct only the most urgent procedures.^19^ One small scale study surveying members of the Texas Association of Otolaryngology found that a majority of their members (47.4%) looked to national bodies such as the American Academy of Otolaryngology-Head and Neck Surgery (AAO-HNS) to provide guidance related to pandemic preparedness in their practice.^20^ The Canadian Society of Otolaryngology (CSO) published a position paper in 2020 to establish guidance for return to practice measures for otolaryngologists, with similar recommendations issued by the American Academy of Otolaryngology-Head and Neck Surgery in February 2021.^21^^,^^22^ Additionally, a living guide exists on the CSO website to aid in clinician decision-making as the pandemic progresses.^19^ There are however limitations in these documents as guidance is typically directed towards physicians without full consideration of the role of allied health professionals, such as Speech-Language Pathologists (SLPs) and audiologists, in provision of policy statements.

To better understand the extensive impact of the COVID-19 pandemic on otolaryngology, we need to assess the impact on individuals beyond physicians and trainees. Furthermore, perspectives and opinions regarding management of otolaryngology services during the COVID-19 pandemic by government and regulatory authorities may guide future policies to alleviate concerns for patients and workers. The objectives of this study are: 1) Longitudinal evaluation of persons undergoing outpatient OHNS surgeries to understand the prevalence and transmission risk of SARS-CoV-2 in this population. 2) Concurrent mixed-methods study of OHNS HCWs to understand the perceptions and fears of working during the COVID-19 pandemic. 3) The impact of PPE usage and patient vaccination status to the perception of safety at work.

## Methods

The study was reviewed and approved by the Conjoint Health Research Ethics Board (CHREB), University of Calgary (approval number REB20-0897) on 10 October 2020. A cross-sectional survey was developed and performed using snowball sampling following distribution to all OHNS societies listed on the International Federation of Otolaryngology Society membership and society groups worldwide.^23^ All consenting OHNS-connected HCWs (physicians, nurses, speech language pathologists, audiologists, technicians, respiratory therapists, and administrative staff) were invited to complete the survey. Sampling was facilitated through the study teams, their affiliates’, and participant’ personal or professional social media accounts (e.g., Twitter, Facebook, Instagram).

Item generation was developed via selection of measures from our existing global perspectives survey with additional measures piloted by clinical context experts, service providers, and researchers. Previously established and validated psychosocial assessment instruments were incorporated into the survey, including the Patient Health Questionnaire-9 (PHQ-9) and Generalized Anxiety Disorder-7 (GAD-7).^24^^,^^25^ The survey was divided into 6-parts: 1) General demographics of the respondents, 2) Employment characteristics of the respondents, 3) Perceptions regarding COVID-19 transmission and infection, 4) Attitudes related to policies and guidelines related to the COVID-19 pandemic, 5) Attitudes and perceptions regarding Personal Protective Equipment (PPE), such as masks and face shields, in the workplace, and 6) Perceptions in the workplace surrounding COVID-19 vaccines. The survey was tested for internal validity and length through a sample population prior to implementation. The survey was distributed to identified members and left accessible from 30 May 2021 to 30 November 2022.

Survey data collected from Qualtrics CoreXM® (Qualtrics International Inc, Seattle, WA), and analyzed in STATA 14.2 (StataCorp, College Station, TX), and reported using descriptive statistics. An a priori recruitment target of 370 participants was set to achieve a 5% margin of error and 95% Confidence Interval to be powered at 80% with an alpha of 0.05.^26^ Geographical distribution of collected responses was reported using Consolidated Standards of Reporting Trials guidelines.^27^ Survey responses were analyzed for representativeness compared to previous otolaryngology surveys conducted during the COVID-19 pandemic.^16^, ^17^, ^18^ Respondents had the option to select “Prefer not to answer” or provide no response for some questions, in order to minimize incomplete surveys. As a result, percentage of responses was computed by removing deficient responses from the denominator to reflect participants’ purposeful responses.

Survey results were summarized using descriptive statistics. Employment roles were categorized according to selection of a primary work role, including surgeon, speech language pathologist, nurse, respiratory therapist, office administration, and others. A chi-square test was conducted for masking and eye protection usage by COVID-19 vaccination status and reported roles within OHNS. Additionally, difference in QoL scores for the five different epidemiological waves of the COVID-19 pandemic^28^ were evaluated using a Chi-Square test. Participant free text responses regarding their perceptions and attitudes to the pandemic impact on their personal and work lives were analyzed for consistent themes. Participant free text responses regarding their perceptions and attitudes to the pandemic impact on their personal and work lives, response from government authorities and OHNS societies were collected. These responses were reviewed and included to highlight predominant themes or responses with quantitative data.

## Results

**Fig. 1:**
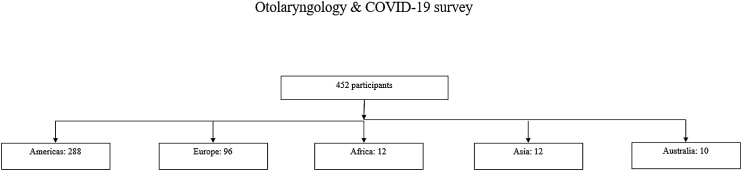
CONSORT diagram of survey respondents and geographical distribution.

A total of 452 participants responded to the survey. A majority (n = 288) of responses were collected from the Americas (Canada, United States, Columbia and Brazil); with 96 responses from Europe, 12 respondents from Africa and Asia, and 10 respondents from Australia (Fig. 1). Respondents were generally representative of previous OHNS surveys conducted during the COVID-19 pandemic.

Our survey included 271 respondents (64.6%) who identified as female and 143 respondents (34.4%) who identified as male (Table 1). The most common roles in OHNS indicated by respondents were surgeons (n = 185, 46.1%), speech-language pathologists (n = 105, 26.2%), and nurses (n = 36, 9%, Table 2). The majority of respondents indicated their primary focus in OHNS as comprehensive or general (n = 105, 26.3%) with a relatively normal distribution of length of time in their indicated role (median = 3–5 years). Most respondents (81.1%) were employed in a full-time (40 h or more) capacity, with work impacted (89.4%) or suspended (81%) due to the pandemic. Additionally, the first wave of the pandemic was identified as being particularly challenging (50.3%) due to fear of illness, managing work-life balance and unclear evidence for restrictions and policies imposed by local health authorities (Table 2).

**Table 1:** General Demographics of survey respondents. Total survey sample size was 452 respondents. Total number of responses may not reach 452 in some instances because respondents elected not to answer the question instead of selecting “Prefer not to answer”.

		n	%
**Age (years)**	18‒25	16	3.8%
	26‒34	115	27.4%
	35‒44	124	29.6%
	45‒54	93	22.2%
	55‒64	50	11.9%
	65‒74	17	4.1%
	75 and greater	4	1.0%
**Sex**	Female	271	64.4%
	Male	145	34.4%
	Prefer not to answer	5	1.2%
**Gender**	Woman	270	64.6%
	Man	143	34.2%
	Non-binary	1	0.2%
	Not listed (other)	4	1.0%
**Living Situation**	Alone in own home	53	12.7%
	In own home with other persons	351	84.2%
	Other	13	3.1%
**Region**	Americas	288	68.9%
	Europe	96	23.0%
	Africa	12	2.9%
	Asia	12	2.9%
	Oceania	10	2.4%
**Persons in household**	1	55	13.4%
	2	121	29.4%
	3	82	20.0%
	4	80	19.5%
	5	45	10.9%
	6	21	5.1%
	7	3	0.7%
	8	1	0.2%
**Marital Status**	Married or in a domestic partnership (common law)	313	75.6%
	Single (never married)	73	17.6%
	Divorced	17	4.1%
	Separated	6	1.4%
	Widowed	5	1.2%
**Ethnicity**	European	223	53.6%
	Asian	68	16.3%
	African	14	3.4%
	Other	2	0.5%
	Prefer not to say	18	4.3%

**Table 2:** Employment characteristics. Total survey sample size was 452 respondents. Total number of responses may not reach 452 in some instances because respondents elected not to answer the question instead of selecting “Prefer not to answer”.

Variable		n	%
Role	Surgeon	185	46.1%
	Speech language pathologist	105	26.2%
	Nurse	36	9.0%
	Other	24	6.0%
	Resident fellow	24	6.0%
	Office assistant	16	4.0%
	Audiologist	7	1.7%
	Medical student	3	0.7%
	Technician	1	0.2%
Length in role (years)	Less than 1	30	7.4%
	1‒2	40	9.9%
	3‒5	92	22.8%
	6‒10	82	20.3%
	11‒15	53	13.2%
	16‒20	35	8.7%
	More than 20	71	17.6%
Employment Status	Employed full time (40 or more hours per week)	326	81.1%
	Employed part time (up to 39-hs per week)	68	16.9%
	Other	8	2.0%
Primary focus in OHNS	Comprehensive/general	105	26.3%
	Laryngology	73	18.3%
	Head and neck surgery	60	15.0%
	Other	52	13.0%
	Not applicable	35	8.8%
	Rhinology	30	7.5%
	Otology	19	4.8%
	Pediatric otolaryngology	17	4.3%
	Facial plastic and reconstructive surgery	5	1.3%
	Sleep surgery	4	1.0%
Work activities affected by COVID-19	Yes	328	89.4%
	No	39	10.6%
Work suspended due to COVID-19	Yes	299	81.0%
	No	70	19.0%
COVID-19 wave that was most challenging to work through	Wave I (Mar 2020‒Jun 2020)	185	50.3%
	Wave II (Sep 2020‒Feb 2021)	67	18.2%
	Wave III (Mar 2021‒Jul 2021)	30	8.2%
	Wave IV (Sep 2021‒Feb 2022)	31	8.4%
	Wave V or beyond (Spring 2022 -)	28	7.6%
	All equally challenging	19	5.2%
	I did not find it challenging	7	1.9%
	Other	1	30.0%
Greatest challenge working during the pandemic	Fear of illness	77	21.1%
	Managing work-life balance	65	17.8%
	Unclear evidence for restrictions and policies	61	16.7%
	Poor outcomes in patients due to delays	41	11.2%
	Changing policies by local bodies	30	8.2%
	Mental health concerns	29	8.0%
	Lack of personal protective equipment	19	5.2%
	Changing models of care (virtual)	15	4.1%
	Financial concerns	14	3.8%
	Other	14	3.8%

Respondents gauged impact the COVID-19 pandemic had on general life beyond workplace concerns through the questions noted in Table 3. The majority of respondents (87%) endorsed the COVID-19 pandemic impacting their life and indicated fearing serious illness of a family member or loved one as their primary concern (52%), which far exceeded the rate of concern for acquiring illness themselves (15.2%) or developing prolonged COVID-19 symptoms (8.4%). Other fears identified to a lesser extent included social isolation, financial impacts, and lack of access to medical care. To better understand perceptions of the pandemic, we assessed respondents’ understanding of SARS-CoV-2 transmission and related evidence, where respondents indicated that COVID-19 transmission occurred primarily via airborne (51%) and droplet/contact (42%). In an interesting contrast, more respondents endorsed high strength of evidence for droplet transmission compared to airborne.

**Table 3:** Perceptions and attitudes regarding COVID-19 infection. Total survey sample size was 452 respondents. Total number of responses may not reach 452 in some instances because respondents elected not to answer the question instead of selecting “Prefer not to answer”.

		n	%
**How do you believe COVID-19 is transmitted?**	Predominantly airborne	181	51.00%
	Predominantly droplet/contact	149	42.00%
	Unsure	16	4.50%
	Predominantly fomite (i.e., surfaces)	4	1.10%
	Other	5	1.40%
**How strong do you believe is the evidence for airborne transmission of COVID-19?**	High	128	36.40%
	Moderate	132	37.50%
	Low	38	10.80%
	Unsure	54	15.30%
**How strong do you believe is the evidence for droplet/contact transmission of COVID-19?**	High	146	41.70%
	Moderate	118	33.70%
	Low	41	11.70%
	Unsure	45	12.90%
**Day-to-day personal life affected by COVID-19**	Yes	320	87.00%
	No	48	13.00%
**Vaccinated for COVID-19**	Yes	391	97.30%
	No	11	2.70%
**Doses of COVID-19 vaccine received**	1	13	3.30%
	2	140	35.90%
	3	225	57.70%
	4	10	2.60%
	5	2	0.50%
**Tested positive for COVID-19**	Yes	137	34.00%
	No	266	66.00%
**Developed COVID-19 symptoms**	Yes	123	89.80%
	No	14	10.20%
**Greatest fear due to COVID-19 pandemic**	Seriou’s illness of a family member or loved one	192	52.00%
	Becoming seriously ill	56	15.20%
	Prolonged illness (long COVID)	31	8.40%
	Regional/global economic crash	14	3.80%
	Social isolation	13	3.50%
	Loss of lives worldwide	12	3.30%
	Loss of finances	11	3.00%
	Mental health concern	11	3.00%
	Lack of access to medical care for you or loved one	8	2.20%
	Increased crime and violence	3	0.80%
	Nothing	3	0.80%
	Loss of employment	2	0.50%
	Loss of relationship (partner)	1	0.30%
	Other	12	3.30%

Self-reported vaccination status of respondents was used to gain a broader understanding of perceptions around COVID-19. Almost all respondents stated they were vaccinated for COVID-19 (97.3%), with a majority inoculated with two or three doses (93.6%). About one-third of respondents indicated having tested positive for COVID-19 (34%); 90% of these people developed symptoms associated with COVID-19, indicating a 10% rate of asymptomatic infections among OHNS-associated HCWs.

When inquired about their perceptions of local health authorities’ responses to and management of the COVID-19 pandemic (Table 4), a majority of respondents agreed with the surgical restrictions imposed (69.7%), though there was incongruence with respondents’ sense of degree of restrictions as less than half reported appropriate scale (43.2%). Similarly, the timing of the restrictions was mostly considered to be too late (49.7%). Although, individuals who completed the survey later were more likely to indicate the timing of restrictions and policies as well-timed (p < 0.001). A clear majority of respondents agreed with policy recommendations and felt supported by OHNS societies (82.1% and 74.9% respectively), though open-field responses indicated that a number of respondents were unaware OHNS societies provided guidance documents.

**Table 4:** Perceptions related to local government and OHNS policies and actions implemented. Total survey sample size was 452 respondents. Total number of responses may not reach 452 in some instances because respondents elected not to answer the question instead of selecting “Prefer not to answer”.

		n	%
**Agreement with surgical restrictions and policies by regional health authorities**	Yes	253	69.7%
	No	110	30.3%
**Timing of restrictions and policies implements by regional health authorities**	Too late	182	49.6%
	Well timed	91	24.8%
	Unsure	77	21.0%
	Too soon	17	4.6%
**Scale of restriction/policies by regional health authorities**	Appropriate	159	43.2%
	Too much	93	25.3%
	Too little	67	18.2%
	Unsure	49	13.3%
**Agreement with policies put forth by OHNS societies**	Yes	298	82.1%
	No	36	9.9%
	Other	29	8.0%
**Support received from OHNS societies**	Yes	265	74.9%
	No	89	25.1%
**Support received from institution/university/management**	Yes	256	70.9%
	No	105	29.1%

**Fig. 2:**
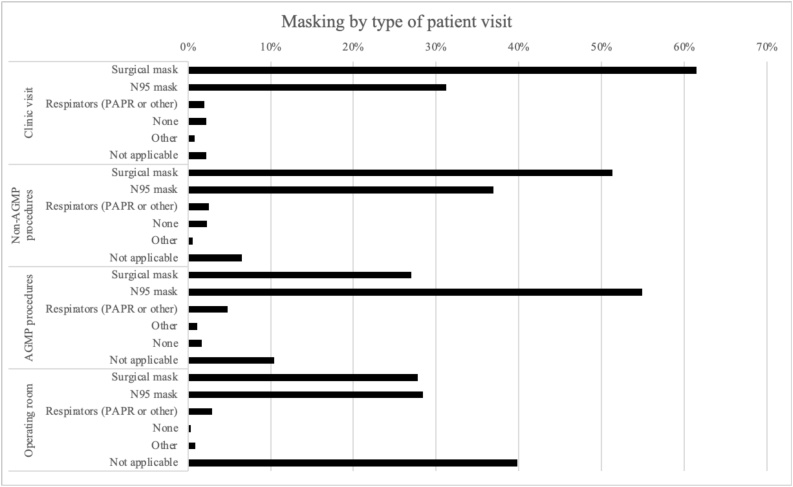
Proportion of respondents reporting form of mask usage by type of patient visit. AGMP, Aerosol-Generating Medical Procedure; PAPR, Powered Air Purifying Respirator.

**Fig. 3:**
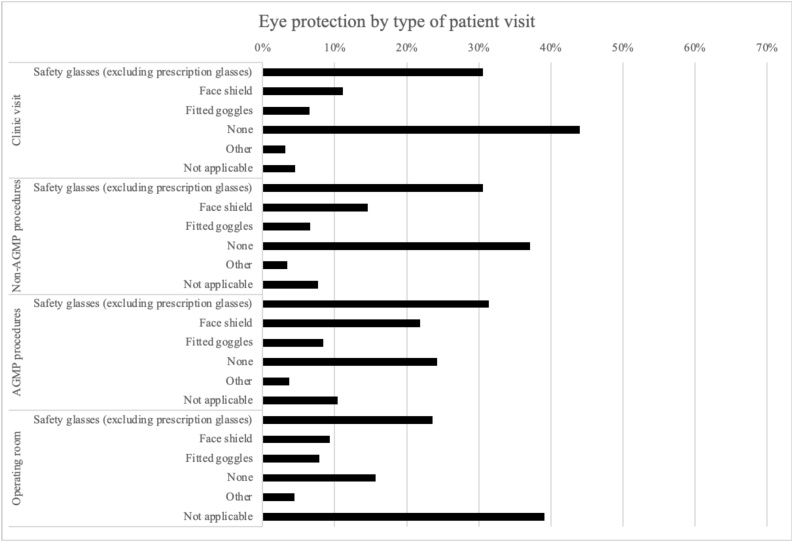
Proportion of respondents reporting form of eye protection usage by type of patient visit.

During outpatient clinic visits, respondents preferred wearing standard surgical masks (61.5%), with trend to higher level protection in mask wearing (such as N95 or respirator) as risk for exposure increased (Fig. 2). A statistically significant association was observed for masking for clinic visits and employment role (p = 0.01) as all respondents that indicated not wearing a mask were surgeons (n = 8). Additionally, 51% of respondents supported mandatory N95 mask usage at their workplace. Eye protection, seen as an important component of infection prevention, had substantially lower uptake than masking for OHNS team members. Respondents most frequently indicated not using eye protection for clinic visits (44%) or non-Aerosol Generating Medical Procedures (AGMP) procedures (37.1%, Fig. 3). Safety glasses were the preferred method of eye protection in all situations, preferred by almost one-third of respondents. An increase in face shield usage was observed as the risk of exposure increased from regular clinic visits to AGMP procedures. No statistical significance was observed for using eye protection and employment role for all types of patient visits and procedures. Finally, an overwhelming majority of respondents (> 95%) utilized both forms of PPE coverage for all visit types. Individuals completing the survey later indicated lower frequency of masking but an increased usage of eye protection. Additionally, there was a statistical difference in adoption of eye protection for office visits, non-AGMP and AGMP procedures for role within OHNS (p < 0.001) with surgeons and residents less likely to report its usage compared to nurses or SLPs. Respondents reported feeling safer at work when vaccinated (83.4%) and if patients were vaccinated (66.1%); about a third of respondents (30.1%) reported indifference to a patient’s vaccination status. We intended to evaluate impact of vaccination status on PPE usage, but only 11 (2.7%) individuals reported being unvaccinated against COVID-19 and therefore statistical analysis was deemed inappropriate. A little more than half of respondents reported implementing pre-operative patient testing as a method to avoid exposure to COVID-19 positive patients (55.9%, Table 5). Respondents indicated a desire for evidence-based guidelines, with 75.9% indicating moderately or very important to incorporate scientific evidence in the policies produced.

**Table 5:** Perceptions of COVID-19 in the workplace. Total survey sample size was 452 respondents. Total number of responses may not reach 452 in some instances because respondents elected not to answer the question instead of selecting “Prefer not to answer”.

		n	%
**Vaccination affecting your feeling of safety at work**	Feel safer	326	83.4%
	No difference	58	14.8%
	Unsure	7	1.8%
**Patients being vaccinated affecting feeling of safety at work**	Feel safer only if fully vaccinated	144	35.8%
	Feel safer even if partially vaccinated	122	30.3%
	No difference	121	30.1%
	Unsure	9	2.2%
	Other	3	0.7%
	Not applicable	3	0.7%
**Routinely test patients pre-operatively for COVID-19**	Yes	195	55.9%
	No	97	27.8%
	Other	57	16.3%
**Current feeling of safety at work from an infection risk standpoint**	Very safe	61	16.6%
	Moderately safe	163	44.3%
	Slightly safe	41	11.1%
	Neutral	35	9.5%
	Slightly unsafe	32	8.7%
	Moderately unsafe	19	5.2%
	Very unsafe	11	3.0%
	Unsure	3	80.0%
	Other	3	80.0%
**Importance of an evidence based surgical pathway for the COVID-19 pandemic (with potential future use)**	Very important	171	49.7%
	Moderately important	90	26.2%
	Slightly important	8	2.3%
	Neutral	41	11.9%
	Not important	7	2.0%
	Other	27	7.8%

## Discussion

The intent of this survey was to evaluate the perceptions and fears of HCWs working during the COVID-19 pandemic in OHNS and OHNS-related fields. Our survey provides new insight into the perception of the COVID-19 pandemic amidst OHNS professionals, with added perspective of a large subgroup of SLP respondents (26.0%) amongst the entire group. While there has been general guidance published for SLP practice in the context of COVID-19,^29^ there has been little integration of clear guidelines for allied health professionals, including SLPs, within current OHNS society guidelines. We observed that our sample had a substantially higher than population rates of COVID-19 vaccine coverage at 97.3%.^30^ We clearly observed this particular frustration in free-form comments in our survey that detailed SLPs feeling a lack of support and lack of focus on ancillary services by OHNS bodies (Supplementary Table 1).

Beyond concerns associated with guidance surrounding the COVID-19 pandemic, respondents generally expressed that the initial wave of the COVID-19 pandemic proved to be the most challenging, which has been previously observed in reports by OHNS medical residents.^31^ The uncertainty of this period was associated with challenges identified by respondents both in the professional and personal sphere. A prominent stressor in the early days of the pandemic in HCWs was fear of infection due to perceived higher exposure risk and susceptibility to the virus.^32^ Interestingly, respondent’s fear of illness of a loved one was especially true if participants indicated being married or in a domestic partnership with 81.2% of respondents indicating it as a primary fear. No additional statements or open field responses provided insight into this perspective, but presumably this relates to the concern of OHNS HCWs either becoming ill and spreading the virus or acting as vectors carrying active virus particles to loved ones. The demographics of our survey contained a little more than half of respondents in the 26–44 years old age groups, with 75.6% self-identifying as being in partnered relationships, which tends to be a demographic inclusive of household members that are younger children. While we did not assess the make-up of respondents’ households, nor vaccination status of household members, COVID-19 vaccines were not available for young children through most of the pandemic. As respondents felt safer when both themselves and patients had received COVID-19 vaccines, it would be interesting to assess if the fear of infecting loved ones was impacted based on vaccination availability for their household members. The survey did not capture what type of vaccines people received, but at the time of survey administration most vaccinated respondents had received mRNA vaccine formulations, although additional vaccines existed in different jurisdictions. Vaccine acceptance and use became a contentious aspect of pandemic management at local and national society levels, but with limited number of people who would have been able to receive a non-mRNA vaccine option we are unable to discern whether the type of vaccine conferred different perceptions of safety to individuals.

Early in the pandemic, a specific source of frustration was public guidance surrounding PPE usage to curb viral spread,^33^ leading to initial distribution shortfalls to HCWs which was subsequently rectified. We found that our survey’s respondents leaned strongly towards implementing both masking and eye protection, with increased levels of protection being used in higher risk-associated procedures. The strong adoption of PPE was likely associated with an increased association with personal safety and guidance from OHNS bodies.^13^^,^^19^^,^^21^^,^^22^^,^^32^^,^^34^

A key component in risk assessment in both personal and systemic pandemic planning of mitigating nosocomial virus spread, is accurate understanding of components of disease dissemination. In our survey, only 16.6% of respondents reported feeling very safe from COVID-19 infection in the OHNS workplace. There was a split in respondents’ opinion of the spread of the SARS-CoV-2 virus, with near equal numbers identifying airborne or droplet/particle spread. Prior studies found HCW understanding of COVID-19 was not significantly different based on role.^35^ Uncertainty surrounding transmission of COVID-19 and its severity may have contributed to the generalized fear of illness within the workplace and at large, as reported in our survey. It is especially important considering emerging data regarding SARS-CoV-2 transmission and evolving health guidelines predicated on the data surrounding transmission during the pandemic was evolving even at the time of this survey. Health jurisdictions initially restricted surgical and procedural activities, out of concern for nosocomial spread as well as the need to manage human resources in key areas. Most respondents agreed with the restrictions imposed in their region, yet overall, there was disagreement in terms of the appropriateness of scale of measures. Less than half of respondents felt the degree of restrictions were appropriate, followed by the response that restrictions were excessive. This is somewhat in contrast with the finding that the greatest number of responses (49%) felt the timing of restrictions and OHNS workplace modifications was too late. Responses submitted later in the timeline considered restrictions to be well-timed compared to earlier survey submissions, which may highlight an evolving perspective and understanding of policies that were implemented.

Overall, this survey provides comprehensive insight into the global impact of the COVID-19 pandemic on OHNS members. The scope is expansive as we gathered opinions beyond professional practice, as well as perceptions regarding COVID-19 transmission. Yet, these findings should be interpreted cautiously as this study was dependent on self-reported data and prone to volunteer bias. The majority of survey responses were collected from Canada and the United States, which may not reflect the opinions and perceptions of OHNS members outside North America. Similarly, with a large proportion of female respondents, there may be additional bias in responses that resonant more with females than males. While our methods attempted to obtain worldwide representation through various invitations, responses were best obtained through snowball sampling and recommendation from colleagues and friends rather than generic invitations. Moreover, this study includes lack of representation from unvaccinated respondents, restricting our ability to conduct statistical analysis regarding PPE usage and vaccination status. Future studies should implement a longitudinal approach to understanding the impact of evolving health guidelines and policies to varying challenges and perceptions over time.

## Conclusion

Individuals working in OHNS reported generalized fear and frustration in their personal and professional lives due to COVID-19 pandemic but exhibited agility to overcome these challenges. Ongoing support from government and OHNS societies, such as frequent communication and consistent guidelines and policies, are key to aid health professionals providing essential care. Incorporating these findings in future pandemic response strategies will help engage members and develop appropriate policies.

## ORCID ID

Charmaine Szalay-Anderson: 0000-0001-9769-6603

Benjamin Ewanchuk: 0000-0001-6197-9222

Ranjani Somayaji: 0000-0003-3731-9675

## Authors’ contributions

Charmaine Szalay-Anderson: Methodology; data curation; original draft preparation.

Benjamin Ewanchuk: data curation; review and editing.

Derrick Randall: Conception; supervision; methodology; data interpretation; formal analysis; review and editing.

Ranjani Somayaji: Conception; supervision; data interpretation; formal analysis; review and editing.

## Sponsor and funding

Department of Surgery Grant (University of Calgary).

## Data availability statement

The authors declare that all data are available in repository.

## Declaration of competing interest

The authors declare no conflicts of interest.
