# Supplementary material for: Impact of the COVID-19 pandemic on individuals working in otolaryngology: a cross-sectional survey
Source: Braz J Otorhinolaryngol. 2026 Jul 22;92(5):101859. doi: 10.1016/j.bjorl.2026.101859 (PMC13425825; doi:10.1016/j.bjorl.2026.101859)
Supplement: Supplementary file 1 [file mmc1.docx]

**BJORL-D-25-00405_Supplementary Material**

**Supplementary Table 1** Survey free text written responses

| **Question** | **Summarized Free Text Responses** |
| --- | --- |
| How safe would you feel at work regarding patient’s vaccine status? | Unsafe because I might contaminate them |
|  | Seeing kids under 5 mostly that are unable to be vaccinated |
| Greatest fear regarding COVID-19? | Social isolation |
|  | Loss of medical and personal autonomy/Critical thinking |
|  | Burnout |
| How has COVID-19 impacted your daily life? | Social isolation |
|  | Mask wearing |
|  | Mental health – guilt and fear |
|  | School closures and home schooling |
|  | Financial concerns |
|  | Death of a family member or friend |
| How has COVID-19 impacted your work? | Continuously changing policies |
|  | Redeployment/Random workload increases due to reduced number of available staff |
|  | Wearing PPE all day |
|  | Too many emails/meetings |
|  | Decreased revenue/Low patient volume |
|  | Cancelled surgeries |
|  | Decreased quality of care to offer patients/Low quality patient interaction via virtual clinic |
| Which COVID-19 wave that was the most challenging? | 1^st^ and 3^rd^ |
| How do you feel about OHNS policies? | Unaware of what the policies were |
|  | Overly cautious policies |
| Did you feel supported by OHNS? | Timely communication and informative resources (guidelines/protocols) |
|  | Needed more focus on ancillary services (SLP)/SLPs did not feel supported |
| Did you feel supported at work? | Timely communication and informative resources (guidelines/protocols) |
|  | Needed more focus on ancillary services (SLP)/SLPs did not feel supported |
|  | Poor distribution of PPE early on but fixed overtime |
|  | Short staffing leading to burnout |
|  | Unable to provide personal input |
|  | Provided with lots of communication and resources |
|  | Not committed to vaccination requirements for staff |
| Different types of masks you use at work? | N95 early on, currently based on patient indication and procedure |
| Different types of eye protection that you use at work? | Mostly prescription eyeglasses |
|  | Mix between face shield (confirmed + patient) or safety glasses (preference) |
| How do you believe COVID-19 is transmitted? | Both airborne and droplet |
| Should N95 masks be mandated? | Depends on virulence in the community |
| Do you pre-test patients for COVID-19? | Only unvaccinated patients or those with unknown status |
|  | Done previously but not anymore |
| How important is an evidence based surgical pathway for you? | Moderately important for continuity of care unless it can be easily adapted for other contaminants |
